# The Young’s Modulus as a Mechanical Biomarker in AFM Experiments: A Tool for Cancer Diagnosis and Treatment Monitoring

**DOI:** 10.3390/s25113510

**Published:** 2025-06-02

**Authors:** Stylianos Vasileios Kontomaris, Anna Malamou, Andreas Stylianou

**Affiliations:** 1 Cancer Mechanobiology and Applied Biophysics Group, School of Sciences, European University Cyprus, 2404 Nicosia, Cyprus; 2 School of Electrical & Computer Engineering, National Technical University of Athens, 157 80 Athens, Greece

**Keywords:** mechanical distributions, cancer diagnosis, combined therapies, contact mechanics, AFM data processing

## Abstract

This review explores recent advances in data processing for atomic force microscopy (AFM) nanoindentation on soft samples, with a focus on “apparent” or “average” Young’s modulus distributions used for cancer diagnosis and treatment monitoring. Young’s modulus serves as a potential key biomarker, distinguishing normal from cancerous cells or tissue by assessing stiffness variations at the nanoscale. However, user-independent, reproducible classification remains challenging due to assumptions in traditional mechanics models, particularly Hertzian theory. To enhance accuracy, depth-dependent mechanical properties and polynomial corrections have been introduced to address sample heterogeneity and finite thickness. Additionally, AFM measurements are affected by tip imperfections and the viscoelastic nature of biological samples, requiring careful data processing and consideration of loading conditions. Furthermore, a quantitative approach using distributions of mechanical properties is suitable for tissue classification and for evaluating treatment-induced changes in nanomechanical properties. As part of this review, the use of AFM-based mechanical properties as a tool for monitoring treatment outcomes—including treatments with antifibrotic drugs and photodynamic therapy—is also presented. By analyzing nanomechanical property distributions before and after treatment, AFM provides insights for optimizing therapeutic strategies, reinforcing its role in personalized cancer care and expanding its applications in research and clinical settings.

## 1. Introduction

**Figure 1:**
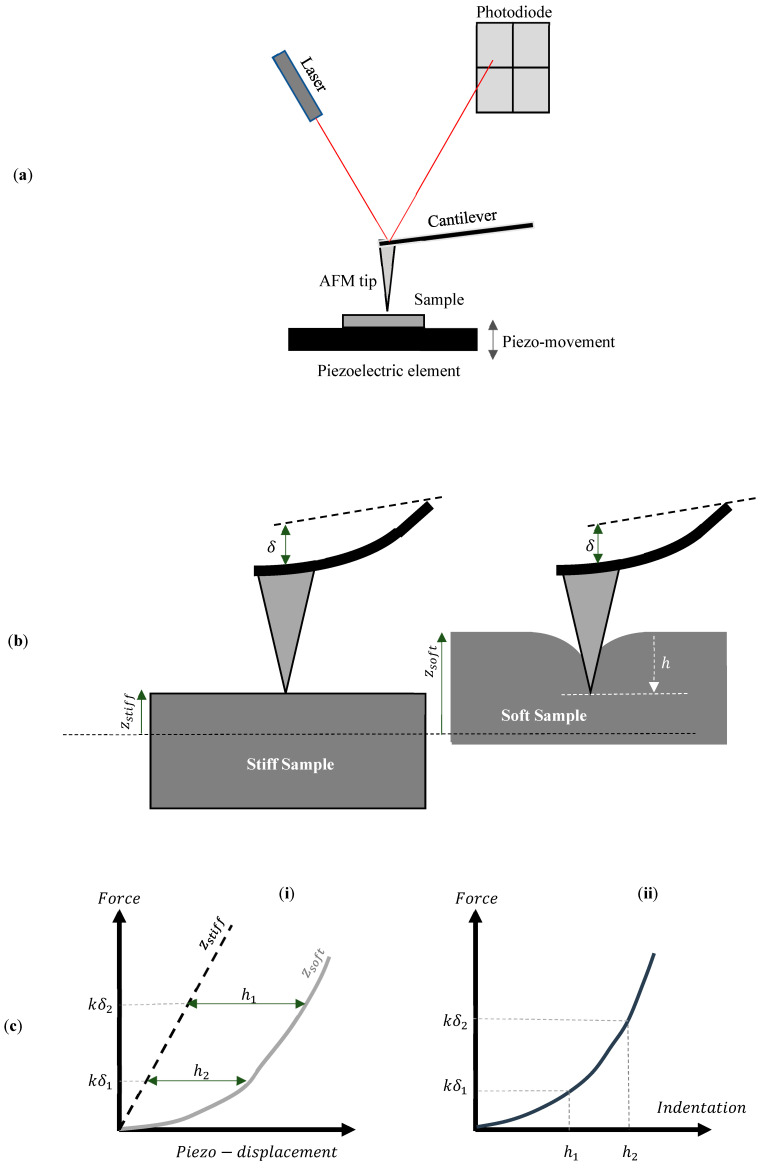
(**a**) A typical AFM system. (**b**) The sample moves toward the AFM tip. In the case of a stiff sample that is not deformed by the AFM tip, the piezo movement is equal to the cantilever’s deflection ($z_{stiff}=\delta$). For a soft sample, the indentation (h) is calculated by subtracting the cantilever’s deflection from the sample’s piezo-displacement ($h=z_{soft}-\delta$). (**c**) (**i**) The force–piezo displacement graph for a stiff and a soft sample. The indentation depth is calculated by subtracting the displacement of the stiff sample from the displacement of the soft sample. (**ii**) The force–indentation curve.

Atomic force microscopy (AFM), introduced in 1986 by Binnig and colleagues, has become a pivotal technique in the field of nanoscale characterization and is a prominent member of the scanning probe microscopy (SPM) family [1,2]. It has become widely used for the nanoscale characterization of various materials, including biological samples, and has established itself as an essential tool in surface science, materials science, and biomedical research [3,4]. AFM employs a sharp, nanometer-sized tip mounted on a cantilever to scan the surface of the sample. The movement of the cantilever is monitored by a laser deflection system, in which a laser beam is directed at the back of the cantilever and reflected onto a position-sensitive photodiode [5,6,7]. The four quadrants of the photodiode measure the beam’s position in both vertical and lateral directions [6]. A typical AFM system is presented in Figure 1a. Cantilever displacement is transformed into force by multiplying it with the spring constant. Piezoelectric elements ensure the precise movement of either the sample or the cantilever. This configuration enables the acquisition of both qualitative data (e.g., imaging) and quantitative data (e.g., Young’s modulus) [5,6,7]. A detailed analysis of the basic components of a typical AFM system is provided in [6,7].

For determining mechanical properties, the AFM tip is pressed into the sample, and nanomechanical characterization is performed by monitoring the cantilever’s deformation as a function of the indentation depth. The nanoindentation at each point generates force–distance (F–z) curves for all areas of interest [7]. The distance (z) represents the piezo-displacement of the sample toward the AFM tip. The goal when performing nanoindentation experiments is to create a force–indentation (F−h) curve at each point of interest. The indentation depth (h) is calculated by subtracting the piezo-displacement ($z_{soft}$) of the soft sample of interest from that of a stiff, non-deformable sample ($z_{stiff}$) for the same deflection of the cantilever (i.e., ${h=z}_{soft}$ − $z_{stiff}$) [7] (Figure 1b,c). Therefore, an essential step in AFM force measurements is the calibration of probe parameters to ensure accurate quantitative results. The force (F) between the tip and the sample is determined using Hooke’s law, which, in the context of AFM, is expressed as F=k·a·V, where k is the cantilever’s spring constant measured in $Nm^{-1}$, a is the Inverse Optical Lever Sensitivity (InvOLS) or deflection sensitivity measured in $nmV^{-1}$, and V is the deflection of the cantilever measured in volts. The photodiode detector of the AFM system directly measures the deflection V. Sensitivity and spring constant calibration are necessary to determine the parameters a and k [8,9,10,11]. The Inverse Optical Lever Sensitivity (a) converts the cantilever’s deflection from volts to nanometers, while the spring constant (k) defines the cantilever’s bending stiffness. A useful guide for the experimental procedures to determine the k and a parameters is presented in [8]. Advanced computations are subsequently conducted to evaluate the various nanomechanical properties, including stiffness and Young’s modulus. These analyses can be carried out using built-in algorithms, plug-ins, or available freeware and commercial software [12,13].

AFM has shown that cancer cells are generally softer than normal cells across various types, including bladder [14,15], breast [16,17], prostate [16,18], oral [19], and glioma [20] tumors. Clinical studies also reveal metastatic cells are softer than benign ones [21], and normal tissue cells follow similar stiffness patterns [9]. Tumor cell stiffness measured by Young’s modulus serves as a diagnostic marker [16]. Additionally, cancer-associated fibroblasts (CAFs) are softer and more invasive than normal fibroblasts [22]. Additionally, studies have shown that malignant and benign cell lines possess distinct viscoelastic properties [23]. In particular, cancer cells exhibit a significantly higher loss tangent than benign cells (the loss tangent is defined as $G^{\prime \prime }/G\prime$ where $G^{\prime \prime }$ is the loss modulus, while $G^{\prime }$ is the storage modulus) [23]. However, it should be noted that cell softening is a response specific to certain cell types [24] and that this characteristic alone cannot be considered a universal indicator of metastatic progression [25].

Therefore, additional research is required to enable AFM to be used effectively as a diagnostic tool for detecting cancer cells and differentiating them from normal cells in clinical practice. Although cancer cells are softer than normal cells, it is widely acknowledged that tumors are stiffer than the surrounding host tissue [26].

The stiffening of tumors is caused by an increase in the extracellular matrix (ECM) composition, primarily due to the accumulation of collagen type I and collagen crosslinking during cancer progression [27,28,29]. Tumor stiffening serves as a key biomechanical feature of solid tumors, which can be detected by both clinicians and patients through palpation. It has been previously shown that AFM can detect unique nanomechanical fingerprints in human breast biopsies, with normal and benign tissues exhibiting uniform stiffness distributions, while malignant tissues displayed two distinct stiffness peaks [26]. Similar bimodal distributions, called the lower elasticity peak (LEP) and higher elasticity peak (HEP), have been observed in human liver tissue and other cancers, including esophageal, renal, colon, and papillary carcinomas [30]. AFM has also been applied to brain tumors for grading based on nanomechanical properties [31]. Clustering algorithms have been developed to classify stiffness measurements into cellular, noncellular, and fibrous tissue components, allowing identification of multiple tissue types [32]. Studies on cervical cancer revealed bimodal Young’s modulus distributions with elevated HEP values in cancerous samples, suggesting HEP as a nanomechanical biomarker [33]. Similar stiffness patterns were found in pancreatic cancer and sarcoma, where AFM aided in monitoring cancer progression and treatment [34,35,36]. Neural networks have been employed for automated identification of brain cancer nanomechanical fingerprints [37]. Collectively, these results support AFM as a valuable technique for early cancer diagnosis, tumor grading, progression tracking, and treatment evaluation [37,38,39,40].

As presented in the previous paragraphs, AFM data can provide valuable information for distinguishing normal and cancerous cells and tissues. However, determining the exact mechanical properties of biological samples at the nanoscale is challenging. These samples often cannot be considered as having infinite depth and are highly heterogeneous, which limits the applicability of classical contact mechanics equations. Over the past two decades, several innovative approaches have been introduced in the literature to enhance the mathematical modeling of AFM force–indentation data for cells and tissues. The purpose of this review is to present these models and offer readers a comprehensive guide to accurately determine mechanical properties at the nanoscale, with a focus on applications related to cancer diagnosis and treatment monitoring. This review paper is organized as follows: Section 2 focuses on Young’s modulus as a physical quantity in cancer research. More specifically, mathematical models for characterizing cells at the nanoscale are presented and discussed. These models account for the sample’s finite thickness and heterogeneity at the nanoscale. Section 3 focuses on experiments on tissues and the mathematical modeling of AFM data. In addition, it presents how the mathematical models discussed in Section 2 can be applied to cancer treatment evaluation. The combination of AFM with photodynamic therapy (PDT) is highlighted as a characteristic example. Section 4 (Discussion and Conclusions) highlights the key aspects of AFM methods in cancer research and discusses their limitations and challenges in clinical applications.

## 2. Utilizing Young’s Modulus as a Biomarker for Cancer Diagnosis: Insights from Classical Contact Mechanics Models

### 2.1. Hertzian Mechanics

Despite the limitations mentioned in the introduction, Young’s modulus remains the most widely used physical quantity for mechanically characterizing soft biological samples, such as cells, at the nanoscale. AFM experiments focused on cancer diagnosis use Young’s modulus as a mechanical biomarker [9,26]. Specifically, the distribution of Young’s modulus across selected nanoregions can offer crucial insights, helping to differentiate between normal and cancerous cells [9]. The key decision for scientists working in AFM mechanobiology and cancer diagnosis is selecting the appropriate model from applied mechanics for data analysis. The most commonly used theory for processing force–indentation data in cell experiments (and generally on biological samples) is Hertzian mechanics [9,41,42]. The procedure involves fitting the force–indentation data to an equation of the following form [9,41,42]:

(1) $$F=ch^{m}$$

where F is the applied force in the sample, h is the indentation depth, and c is a constant parameter that depends on the material properties and the geometry of the indenter, while m is a constant parameter that depends only on the indenter’s properties. For conical indenters, $c=\frac{2}{\pi }\frac{Ε}{1-v^{2}}\tan\left(\theta \right)$, where E, v are the Young’s modulus and the Poisson’s ratio of the tested sample, θ is the cone’s half angle, and m=2 (the c and m parameters for conical indenters were originally derived by Sneddon in 1965 [43]). For parabolic indenters, $c=\frac{4}{3}\frac{E}{1-v^{2}}R^{1/2}$, where R is the indenter’s radius and m=3/2. Classic pyramidal indenters are usually approximated as perfect cones in AFM indentation experiments [9,42,44]. However, an alternative equation for a four-sided symmetrical pyramid also exists. In this case, $c=\frac{1}{\sqrt{2}}\frac{Ε}{1-v^{2}}\;\tan\left(\theta \right)$ [45]. It is also important to note that the Hertzian equation can be used for spherical indenters at small indentation depths compared to the tip radius (h<<R) [46]. A typical limit in the literature is h<R/10 [47,48]. For larger spherical indentations, Sneddon’s equations should be used instead [43,49]:

(2) $$F=\frac{E}{2\left(1-v^{2}\right)}\left[\left(r_{c}^{2}+R^{2}\right)ln\left(\frac{R+r_{c}}{R-r_{c}}\right)-2r_{c}R\right]$$

The indentation depth is related to the contact radius ($r_{c}$) with the following equation [43,49]:

(3) $$\ln\left(\frac{R+r_{c}}{R-r_{c}}\right)=\frac{2h}{r_{c}}$$

However, Equations (2) and (3) do not provide a direct relationship between the applied force and the indentation depth. Therefore, a straightforward equation has been recently developed to simplify the fitting procedure [50]:

(4) $$F=\frac{4ER^{\frac{1}{2}}}{3\left(1-v^{2}\right)}h^{\frac{3}{2}}Z$$

where [50]

(5) $$Z=c_{1}+\sum_{M=2}^{N}\frac{3}{2Μ}c_{M}R^{\left(\frac{3}{2}-M\right)}h^{M-3/2}$$

For h<R (which is usually the case), N=3 and $c_{1}$ = 1.014, $c_{2}=-0.09059$, and $c_{3}=-0.09431$ [50]. Therefore,

(6) $$Z=c_{1}+\frac{3}{4}c_{2}R^{-\frac{1}{2}}h^{\frac{1}{2}}+\frac{3}{6}c_{3}R^{-\frac{3}{2}}h^{\frac{3}{2}}$$

Other approaches for deep spherical indentations have been also presented in [51].

### 2.2. The Finite Thickness of the Sample

The equations presented in Section 2.1 assume that the sample behaves as a linear elastic half-space. There are two main drawbacks to this assumption. The first drawback is related to the finite thickness of the sample. The first work aimed at correcting the Hertz equations for parabolic indenters when testing thin samples was presented by Dimitriadis et al. [52]. Following this, Gavara and Chadwick presented the first equations for conical indenters [53]. Alternative equations were proposed by Santos et al. [44]. The most recent attempt to provide accurate equations for various indenter geometries was presented by Garcia and Garcia [54]. A common feature of all the equations mentioned above is that they multiply the result given by the classic Hertzian equation by a polynomial function f(h,H), which depends on the indentation depth, the sample’s thickness, the indenter’s properties, and whether the sample is bonded to the substrate or not. At this point, it should be noted that the term “Hertzian equation” is not limited to the case of parabolic geometry of the indenter; it is a generic term that also includes any geometry, such as Sneddon’s equation for perfect conical indenters [43]. Therefore,

(7) $$F=F_{Hertz}f\left(h,H\right)$$

According to Garcia and Garcia’s work, for cells bonded to the substrate and for parabolic indenters [54],

(8) $$F_{\mathrm{paraboloid}}=\frac{16}{9}ER^{\frac{1}{2}}h^{\frac{3}{2}}\left[1+\frac{1.133\sqrt{Rh}}{H}+\frac{1.497Rh}{H^{2}}+\frac{1.469{\left(Rh\right)}^{\frac{3}{2}}}{H^{3}}+\frac{0.755{\left(Rh\right)}^{2}}{H^{4}}\right]$$

In addition, for conical indenters [54],

(9) $$F_{cone}=\frac{8}{3\pi }E\tan\left(\theta \right)h^{2}\left[1+\frac{0.721h\tan(\theta )}{H}+\frac{0.650h^{2}\tan^{2}\left(\theta \right)}{H^{2}}+\frac{0.491h^{3}\tan^{3}\left(\theta \right)}{H^{3}}+\frac{0.225h^{4}\tan^{4}\left(\theta \right)}{H^{4}}\right]$$

In both Equations (8) and (9), it was assumed that the Poisson’s ratio is v=0.5 due to the high water content when testing biological cells under real conditions [53,54]. It should be noted that most of the polynomial corrections in the literature, regardless of the exact shape of the AFM tip or whether the sample is bonded to the substrate, can be presented in the following form [44,52,53,54]:

(10) $$f\left(h,H\right)=1+\sum_{n=1}^{4}c_{n}{\left(\frac{r}{H}\right)}^{n}$$

where, r is a characteristic length that depends on the indenter’s shape. For example, $r=\sqrt{Rh}$ for parabolic (this is equal to the contact radius between the indenter and the sample [46]) and r=htan(θ) for conical indenters [53,54]. The coefficient $c_{n}$ depends on both the indenter’s properties and on whether the sample is bonded to the substrate.

### 2.3. The Heterogeneity of Biological Samples

**Figure 2:**
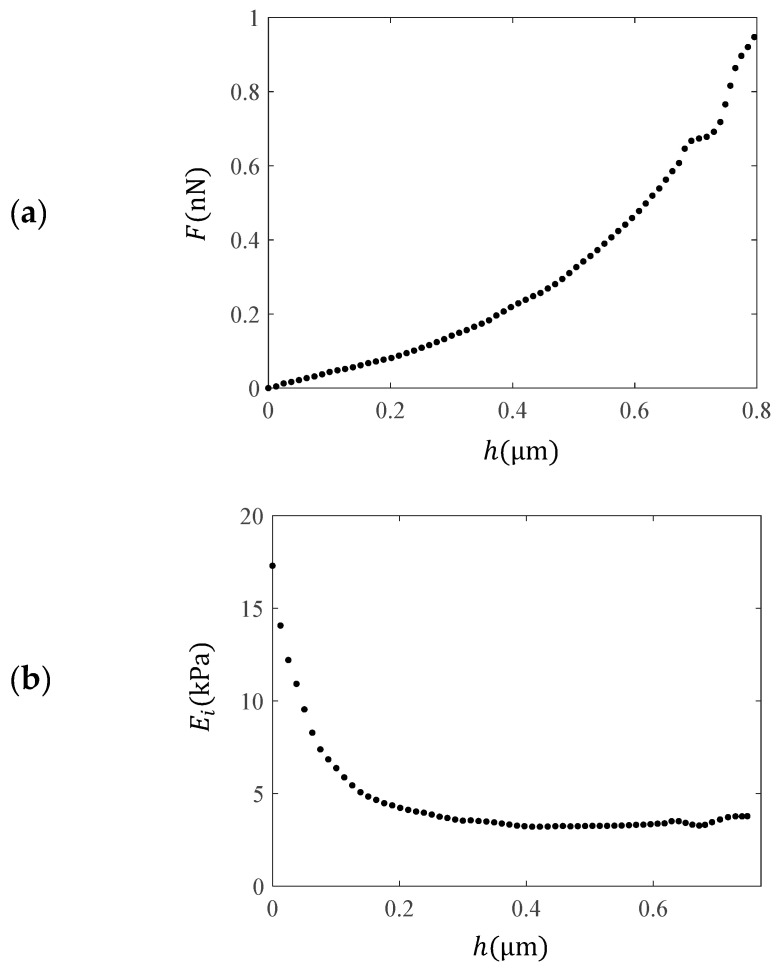
Experimental data using AFM on a fibroblast. (**a**) Force–indentation data. (**b**) Using each pair of $h_{i}$ and $F_{i}$ data, an “apparent” point-by point modulus was calculated, named as the “pointwise modulus $\left(E_{i}\right)$”. The calculation was performed using the AtomicJ software [12].

The second drawback of using the classic Hertzian equations for data processing is related to the heterogeneity of biological samples [41]. Several interesting attempts have been presented in the literature to derive new equations that can reliably approximate the real mechanical behavior of a single cell. The oldest and simplest method is based on the calculation of the “pointwise” modulus (i.e., the apparent point-by-point modulus) [55,56,57]. This is based on using each pair of force–indentation values ($h_{i}$ and $F_{i}$), leading to a “pointwise modulus ($E_{i}$)” for each pair [55,56,57]. A typical example is shown in Figure 2. In Figure 2a, force–indentation data collected from a fibroblast is displayed. The AFM tips were approximated as perfect cones, as the indentation depth was significantly greater than the tip radius (nominally 20 nm). The cone’s half-angle was 25°, and the nominal spring constant was 0.01 N/m [12]. Figure 2b shows the modulus values at each indentation depth. The force–indentation data for the fibroblast presented in Figure 2 was retrieved from the AtomicJ repository [12]. The protocol related to the experiments on fibroblasts is presented in [12].

The “pointwise modulus” approach is an “empirical” method used to test a soft sample’s (e.g., a cell’s) heterogeneity. In addition to this approach, more rigorous methods have also been developed. Ding et al. proposed an approach in which the calculated Young’s modulus is influenced by surface tension effects [58]. Their study establishes two straightforward yet robust relationships between force and indentation depth for spherical and conical indentations, accounting for surface tension effects. Through dimensional analysis and finite element simulations, it was shown that classical contact mechanics theories overestimate the elastic modulus when the indentation depth is smaller than the intrinsic length, defined as the ratio of surface tension to elastic modulus. In particular, when using parabolic indenters [58],

(11) $$F=\frac{4}{3}\frac{E}{1-v^{2}}R^{1/2}h^{3/2}\left[1+a_{s}{\left(\frac{s}{\sqrt{Rh}}\right)}^{\beta_{s}}\right]$$

where, $s=2\gamma (1-v^{2})/E$, γ is the surface energy density, $a_{s}=0.88\pm 0.0037$, and $\beta_{s}=0.87\pm 0.0034$. In addition, for conical indenters [58],

(12) $$F=\frac{2}{\pi }\frac{E}{1-v^{2}}\tan(\theta )h^{2}\left[1+a_{C}{\left(\frac{s}{htan(\theta )}\right)}^{\beta_{C}}\right]$$

where, $a_{C}=0.95\pm 0.0097$ and $\beta_{C}=0.92\pm 0.0088$.

Therefore, the aforementioned model suggests that, when performing a classic fitting procedure, the determined parameter (this is usually mentioned in the literature as the “apparent” Young’s modulus) should be

(13) $$E_{a}=E\left[1+\lambda \left(h\right)\right],\;\lambda \left(h\right)>0\;$$

where, λ(h) is a monotonically decreasing function. Thus, the calculated Young’s modulus of the cell is overestimated in conventional data processing methods, and the determined value approaches the true modulus (E) for h≫s. This model is in good agreement with many different studies presented in the literature [24,59].

Another approach for materials with strongly depth-dependent behavior has been presented by Kontomaris et al. [60]. In this case, the material is considered as the sum of N homogeneous slices with N→∞. Each slice is considered homogeneous and isotropic. It has been proven that the apparent Young’s modulus when using the classic Hertzian equations is the average Young’s modulus of these slices [60]. The idea can be briefly explained using the weighted mean value theorem for integrals [61]. In particular, let f,  g:  [0, h] → R be such that f is continuous and g is integrable and does not change the sign on [0, h]. Then, there exists a number c ∈ (0, h) such that

(14) $$\int_{0}^{h}f\left(x\right)g\left(x\right)dx=f(c)\int_{0}^{h}g\left(x\right)dx$$

Therefore, regardless of the exact shape of the indenter, using the generic differential equation [62,63] involving the force applied to a material with respect to the indentation depth, the following conclusion is drawn [60,61]:

(15) $$\frac{dF}{dh}=\frac{2}{1-v^{2}}E\left(h\right)r_{c}\left(h\right)\Rightarrow F=\frac{2}{1-v^{2}}\int_{0}^{h}E\left(y\right)r_{c}\left(y\right)dy\Rightarrow F=\frac{2}{1-v^{2}}E\left(c\right)\int_{0}^{h}r_{c}\left(y\right)dy\;\;$$

where, $E\left(c\right)$ is an “average” value representing the mechanical properties of the tested material over the domain 0≤y≤h. In addition, the integral of the $r_{c}\left(y\right)$ function depends on the shape and size of the indenter and the indentation depth. For example, for parabolic indenters $r_{c}\left(y\right)=Ry,$ therefore,

(16) $$\int_{0}^{h}r_{c}\left(y\right)dy=\frac{2}{3}Rh^{3/2}$$

**Figure 3:**
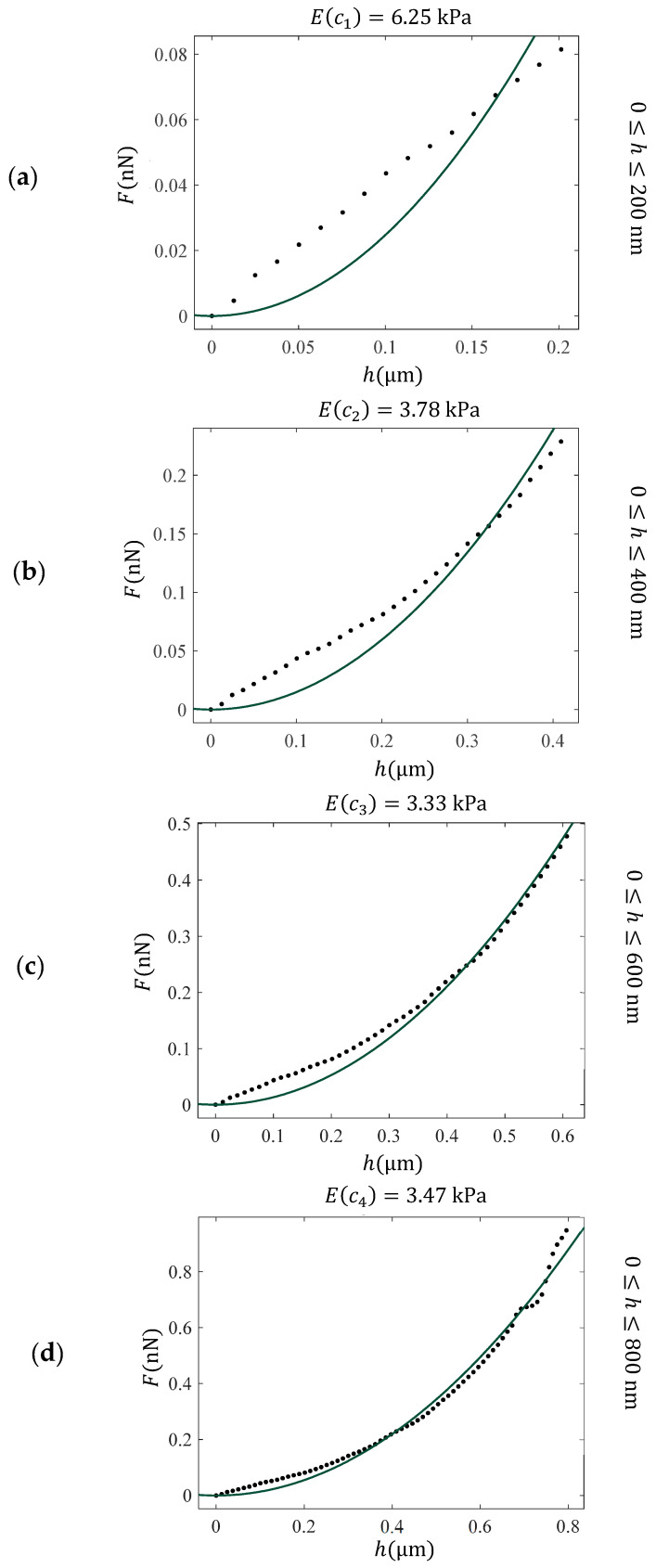
Fitting of force indentation data obtained on a fibroblast to the classic Sneddon’s equation for conical indenters (Equation (1) with $c=\frac{2}{\pi }\frac{Ε}{1-v^{2}}\tan\left(\theta \right)$ and m=2) for different domains. (**a**) 0≤h≤200 nm, (**b**) 0≤h≤400 nm, (**c**) 0≤h≤600 nm, and (**d**) 0≤h≤800 nm. The Young’s modulus was calculated as a fitting parameter and resulted in 6.25 kPa (**a**), 3.78 kPa (**b**), 3.33 kPa (**c**), and 3.47 kPa (**d**). These values can be used to derive the “average” Young’s modulus with respect to the indentation depth function. The “average” Young’s modulus, *E*(*c*), provides a more accurate assessment of the depth-dependent mechanical response of heterogeneous materials.

Experiments on normal and cancerous cells have shown that performing various fittings for different indentation depths $h_{1},\;h_{2},\ldots ,\;h_{N}$ on the data leads to N values of E(c) parameters, denoted as $E(c_{1})\;$, $E\left(c_{2}\right),\ldots ,\;E\left(c_{N}\right)$ [60]. A characteristic example is presented in Figure 3. The force–indentation data presented in Figure 2a are fitted to the classic Sneddon’s equation in the following domains: 0≤h≤200 nm, 0≤h≤400 nm, 0≤h≤600 nm, and 0≤h≤800 nm. As clearly presented in Figure 3, the mechanical properties of the fibroblast vary significantly with depth due to structural complexity. Traditional models, such as those of Sneddon or Hertz, which assume homogeneity and linear elasticity, fail to capture this variability—particularly at shallow indentation depths. This limitation motivates the use of depth-dependent approaches, such as the “apparent” or “average” Young’s modulus E(c), which provide a more accurate representation of the material’s mechanical behavior by accounting for variations across different indentation depths.

Using the E(c) with respect to h data, the E=f(h) function can be determined. Using multiple experiments on cells, it was shown that for cells, the “apparent” or “average” Young’s modulus is related to the indentation depth with the following generic function [64,65]:

(17) $$E\left(c\right)=Ah^{\xi }+C$$

where A>0,  C>0, and ξ<0. Equation (17) is in perfect agreement with Equation (13), as proposed by Ding et al.’s model [58].

At this point, it is important to note that AFM nanoindentation of membrane-enclosed samples often shows an initial linear force–indentation regime, leading to inaccurate Young’s modulus values when this region is included in the fit (see, for example, Figure 3a,b). Weighting both poor fits in the initial phase and better fits at deeper indentations equally may introduce systematic bias, causing samples with similar bulk elasticity but different initial responses to appear different. Careful selection of the fitting range can improve the accuracy of mechanical characterization.

**Figure 4:**
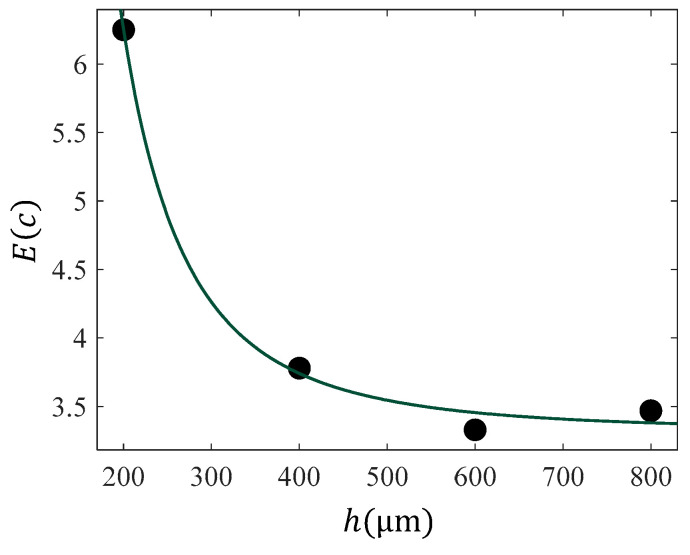
The E(c) data with respect to indentation depth for the case of data presented in Figure 2. The data was fitted to Equation (17) with $A=8.134\times 10^{6}\;kPa\cdot nm^{2.8},\xi =-2.8$, and C=3.321 kPa. The R-squared coefficient resulted in $R_{s.c.}^{2}=0.9955$.

In Figure 4, the results for the “average” or “apparent” Young’s modulus, presented in Figure 3, are plotted and fitted to Equation (17). The fitting coefficients resulted in *A* = $8.134\times 10^{6}\;kPa\cdot nm^{2.8},\xi =-2.8$, and C=3.321 kPa. The R-squared coefficient resulted in $R_{s.c.}^{2}=0.9955$. This procedure can be also extended for any indenter’s geometry [66].

The procedure described above (i.e., the calculation of the “apparent” Young’s modulus with respect to indentation depth) can be performed in various nanoregions, leading to a set of mechanical property maps. This set can reveal the sample’s heterogeneity along the x, y, and z axes. More specifically, this procedure includes different indentation depths, as explained below for the simple case of an i x j map set. For indentation depth $h_{N}$, the Young’s modulus values are recorded across the sample surface as a spatial map $\left\{E_{ij}\right\}$, where each $E_{ij}$ corresponds to the modulus at the position indexed by i and j.

In addition, for indentation depth ${h\prime }_{N}$, the Young’s modulus values are recorded across the sample surface as a spatial map $\left\{{E\prime }_{ij}\right\}$, where each ${E\prime }_{ij}$ corresponds to the modulus at the position indexed by i and j, and so on.

**Figure 5:**
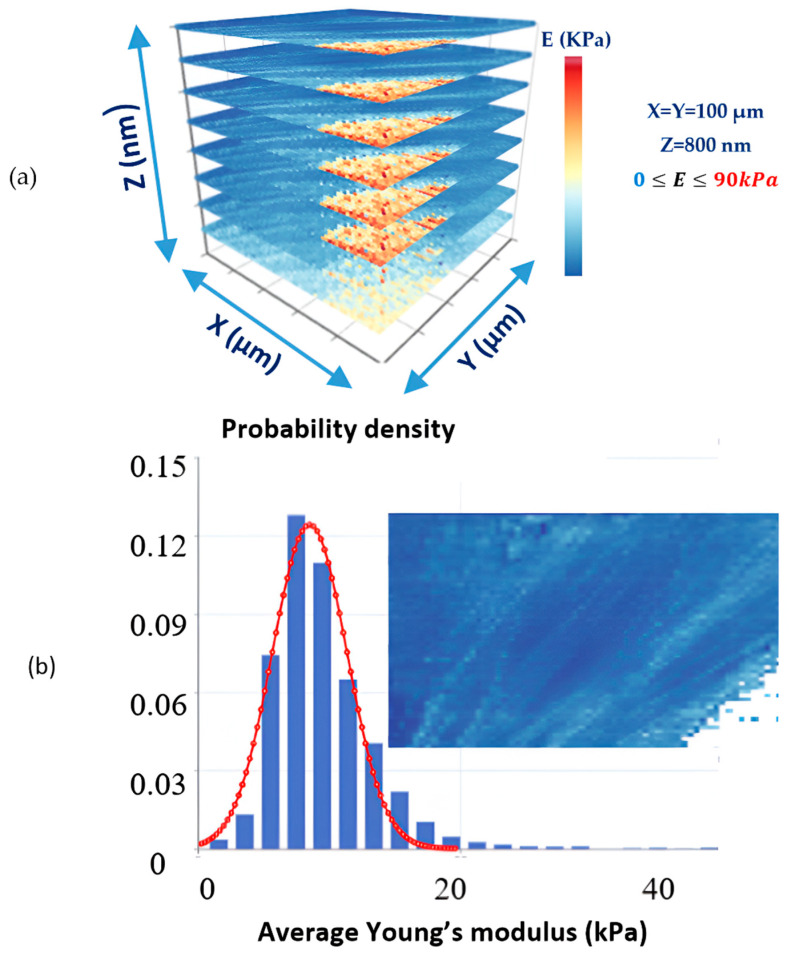
(**a**) Eight “average” Young’s modulus maps of a fibroblast reveal its depth-dependent mechanical properties. The x−y dimensions of each map were 100 μm, and the maximum indentation depth was 800 nm. (**b**) An “average” Young’s modulus map and the distribution of average E-values. The values on the bottom right side of the map have been excluded due to the strong influence of the substrate. The protocol used to obtain the AFM indentation data is presented in reference [12], and the data processing method is described in reference [65].

Apparent Young’s modulus values are displayed in the form of typical Young’s modulus maps, as commonly presented in the literature [59,67]. By combining these maps, a 3D mechanical characterization of the sample of interest can be obtained [65]. A typical example is presented in Figure 5a, where eight “average” Young’s modulus maps are combined to provide a complete 3D characterization of a fibroblast [65]. The protocol used to obtain the AFM indentation data is presented in reference [12], and the data processing method is described in reference [65]. In Figure 5b, a typical average Young’s modulus map is presented along with the average E—distribution. Due to the strong influence of the substrate on the bottom right part of the map, the corresponding values have been removed.

In addition, it has been recently shown that this approach can be also extended for heterogeneous samples with finite thickness, which is the most accurate representation of a real biological cell [68]. For example, the following model was proposed for a conical indenter [68]:

(18) $$F_{cone}=\frac{8}{3\pi }E\left(c\right)tan\left(\theta \right)\left[1+\sum_{n=1}^{4}c_{n}{\left(\frac{r}{H}\right)}^{n}\right]$$

When using Equation (18) for data processing, the “apparent” Young’s modulus can be described by the general Equations (13) and (17).

In addition, Chen et al. introduced a robust approach known as the trimechanic theory, which addresses the elastic behaviors of soft materials and biomaterials under different conditions [69]. Variations in elastic behaviors indicate changes in the material’s context. The trimechanic theory allows for the quantification of elastic response differences by combining three nanomechanical actions, each governed by constant, linear, and non-linear forces, respectively [69]. This theory naturally extends Sneddon’s pyramid model to investigate elastic heterogeneity [69]. The depth-dependent mechanical properties of any soft biological sample can be equally determined using this approach.

### 2.4. Tip Shape-Related Pseudo-Softening Behavior

An important issue affecting the accurate mechanical recording of the tested soft sample is the imperfect conical shape of classic AFM pyramidal tips. More specifically, there is an error associated with the tip apex. Consider, for example, the generic case of a conical indenter with an arbitrarily shaped tip apex of the form $f\left(r\right)=Br^{n}$, where B and n are positive constants, and r is the radius at the contact depth between the tip and the sample [62,63]. In this case, the force applied on the sample is related to the indentation depth, as below [70]:

(19) $$F=\frac{2}{\pi }\frac{E}{1-v^{2}}\tan\left(\theta \right)h^{2}+\frac{4}{\pi }\frac{E}{1-v^{2}}\left\{\frac{1}{{\left(\sqrt{\pi }Β\right)}^{1/n}}{\left[\frac{\Gamma \left(\frac{n}{2}+\frac{1}{2}\right)}{\Gamma \left(\frac{n}{2}+1\right)}\right]}^{1/n}\;h_{T}^{1/n}-h_{T}\tan\left(\theta \right)\right\}h$$

where, Γ is the gamma function, θ is the half-angle of the conical parts of the indenter, and $h_{T}$ is the transition depth between the conical and the spherical parts of the indenter. For the case of a sphero-conical indenter, Equation (19) simplifies as below [70]:

(20) $$F=\frac{2}{\pi }\frac{E}{1-v^{2}}\tan\left(\theta \right)h^{2}+\frac{4}{\pi }\frac{E}{1-v^{2}}[R_{T}-\tan\left(\theta \right)h_{T}]h\;\;$$

where, $R_{T}$ is the radius at the transition depth. Finally, for a truncated cone [70],

(21) $$F=\frac{2}{\pi }\frac{E}{1-v^{2}}\tan\left(\theta \right)h^{2}+\frac{4}{\pi }\frac{E}{1-v^{2}}R_{T}h$$

The generic form of Equations (19)–(21) is the sum of Sneddon’s equation for conical indenters and a term related to the tip shape, denoted as Φ(h) (where Φ(h)>0):

(22) $$F=\frac{2}{\pi }\frac{E}{1-v^{2}}\tan\left(\theta \right)h^{2}+\Phi (h)$$

Equation (22) is the exact equation for non-perfect conical indenters. The correction term Φ(h) accounts for imperfections at the tip apex. Although real pyramidal indenters are typically modeled as conical, tip apex imperfections can lead to significant errors in Young’s modulus calculations. Therefore, the physical meaning of the correction function Φ(h) is to compensate for the actual geometry of the tip apex in real indentation experiments [70].

Therefore, if the classic Sneddon’s equation is used to fit the data, it will lead to an overestimation of the apparent Young’s modulus, since

(23) $$\frac{2}{\pi }\frac{E_{Sneddon}}{1-v^{2}}\tan\left(\theta \right)h^{2}=\frac{2}{\pi }\frac{E}{1-v^{2}}\tan\left(\theta \right)h^{2}+\Phi (h)\Rightarrow E_{Sneddon}=E+\frac{\pi }{2}\frac{1-v^{2}}{\tan\left(\theta \right)h^{2}}\Phi (h)$$

where, $E_{Sneddon}$ is the “apparent” Young’s modulus calculated using Sneddon’s model. It is obvious that $E_{Sneddon}>E$. Therefore, the “apparent” Young’s modulus is overestimated when the indenter is considered a perfect cone, even if the sample is purely homogeneous and isotropic.

### 2.5. The Oliver–Pharr Method

A key advancement in data processing for nanoindentation experiments was the development of a straightforward formula describing the interaction between a rigid, axisymmetric indenter and an elastic half-space [62]. This formula was rigorously derived by Pharr, Oliver, and Brotzen in 1992. Specifically, they demonstrated that the Young’s modulus can be determined using the following equation [62]:

(24) $$E=\frac{\sqrt{\pi }}{2}\left(1-v^{2}\right)\frac{S}{\sqrt{A}}$$

In Equation (24), S represents the contact stiffness of the sample and is calculated as the derivative of the force–indentation curve at the maximum indentation depth $\left(S={\left.\frac{dF}{dh}\right|}_{h_{max}}\right)$ [63]. Additionally, A denotes the projected contact area of the indenter at the contact depth [63]. For parabolic indenters (or spherical indenters for h<<R) [46],

(25) $$A=\pi Rh_{max}$$

For spherical indenters and large indentation depths [46],

(26) $$A=\pi \sqrt{2Rh_{c}-h_{c}^{2}}$$

where $h_{c}$ is the contact depth. For h<<R, $h_{c}=h_{max}/2$ and Equation (26) reduces to Equation (25). For conical indenters,

(27) $$A=\pi h_{c}^{2}\tan^{2}\left(\theta \right)$$

where $h_{c}=2h_{max}/\pi$ [46].

For purely elastic contact, the classic Hertzian and Sneddon equations presented in Section 2 conform to the general form of Equation (24), as this equation applies to any axisymmetric indenter. For example, by substituting Equation (25) into Equation (24), the classic Hertzian equation for parabolic indenters can be easily derived [46]. Similarly, the classic Sneddon equation for conical indenters can be readily obtained by substituting Equation (27) into Equation (24) [46]. It is important to note that the classic Oliver and Pharr approach considers elastic–plastic contact, and the unloading force–indentation curve is used for data processing [63]. For a purely elastic contact, however, the loading and unloading curves are identical, and using Equation (24) is equivalent to using Hertz’s or Sneddon’s equations. Nevertheless, for elastic–plastic contact, it is essential to use the unloading force curve for Young’s modulus determination [63].

Equation (24) was also used by Kontomaris and Malamou in [50] to derive Equations (4)–(6) for deep spherical indentations. In addition, previous research generalized Equation (24) for heterogeneous samples [60,66]. Equation (24) was used to generalize the classic Hertzian and Sneddon equations for heterogeneous samples (see Equation (15)), which can be considered as the sum of N homogeneous slices, where N→∞, under the condition that two successive slices have similar mechanical properties [60]. The models presented in Section 2.1, Section 2.2, Section 2.3, Section 2.4 and Section 2.5 are summarized in Table 1.

### 2.6. Viscoelasticity

It is also important to note that, in many instances, differences are observed between the loading and unloading curves for biological samples, with the two curves not being identical. This discrepancy arises due to the viscoelastic nature of biological materials. Viscoelasticity occurs when a material exhibits both elastic and viscous properties [71]. The term “viscous” refers to the material’s tendency to deform slowly under an external force, while “elastic” indicates that the material returns to its original shape once the load is removed. For purely elastic materials, the loading and unloading curves coincide. However, in viscoelastic materials, a “hysteresis” loop is formed, with the area within the loop representing the energy lost and dissipated as heat [71]. Moreover, the mechanical properties of viscoelastic materials depend on the deformation rate [72]. Specifically, the material’s stiffness increases as the loading rate increases, resulting in a family of load-indentation curves, each representing the sample’s mechanical properties at a different loading rate [70]. The viscoelastic behavior of biological samples is attributed to the aqueous environment in which they are tested, as these materials are typically examined in liquid conditions to replicate physiological settings.

Viscoelastic behavior implies that the measured elastic moduli are affected by the frequency at which the cantilever is ramped. The relationship between the elastic modulus and probing frequency adheres to a weak power law, with exponents ranging from 0.10 to 0.25 [73,74,75]. Although this was not a significant concern in the past, modern AFMs are now capable of obtaining force curves across a much broader range of ramping frequencies, up to 2 kHz [6]. Therefore, it is crucial to consider frequency-dependent effects when comparing newly published data obtained at high ramping frequencies with older data collected at frequencies below 1 Hz. Therefore, to minimize viscoelastic effects, an appropriate small ramp frequency is often used [6,72,75].

This review focuses on approximating the behavior of cells and tissues as elastic by considering low loading rates. The reason for this approach is that it is likely the simplest method, which can easily lead to reproducible results (since, as mentioned earlier, mechanical properties vary significantly at high loading rates). This is why many major papers related to cancer diagnosis protocols approximate the sample as elastic [26]. Readers seeking the classic literature on the viscoelasticity of soft materials may refer to the following references [72,73,74,75,76,77,78,79,80,81,82,83,84,85,86,87,88,89,90,91].

## 3. Modeling Results from Normal, Benign, and Cancer Tissues

### 3.1. AFM as a Diagnostic Tool

**Figure 6:**
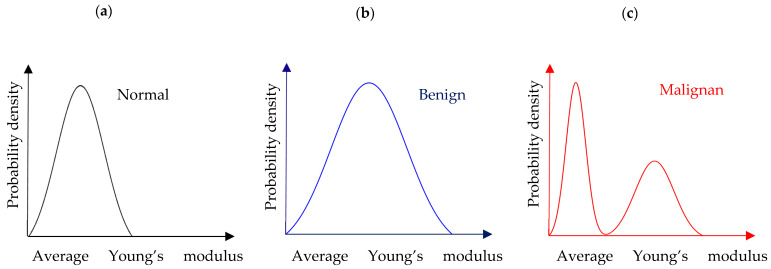
(**a**) A probability density histogram showing the “average” Young’s modulus distribution of a normal tissue section. (**b**) The case of a benign tissue section. (**c**) The case of a malignant tissue section.

AFM enables the detailed mechanical characterization of tissue at the nanoscale, providing information on properties such as stiffness and elasticity that correlate with pathological changes. By analyzing biopsy samples, AFM can detect subtle differences between normal, benign, and malignant tissues, offering a sensitive and objective method for cancer diagnosis. This capability positions AFM as a promising complementary tool alongside traditional histopathology, potentially improving diagnostic accuracy and enabling earlier detection of cancer. Cancer progression alters the mechano-cellular phenotype, affecting the tumor microenvironment (TME). AFM has enhanced biological specimen characterization, aiding cancer diagnosis and prognosis [26,30,31,34,36,92,93,94]. AFM studies show that cancer cells are “softer” due to cytoskeletal changes, as already mentioned, while cancerous tissues are stiffer due to increased extracellular matrix (ECM) proteins, mainly collagen type I [29]. As a result, AFM is valuable for monitoring mechanical changes at both cellular and tissue levels [26]. A noteworthy quantitative approach for distinguishing normal from cancerous tissues has been previously introduced [26,30,92]. The idea is to plot frequency histograms of the measured Young’s modulus values, which should be referred to as “apparent” or “average” Young’s modulus values for the reasons discussed in Section 2. Thus, the graph of the probability with respect to the average elastic modulus for tissues can be delivered. The probability provides the occurrences of a single “average” Young’s modulus value expressed as a fraction of 100. The overall procedure is presented in Figure 6. For normal or benign tissue, the distribution of stiffness typically follows a single-peak pattern, as shown in Figure 6a,b. In benign tissue, this peak may appear broader (Figure 6b). In contrast, cancerous tissue exhibits a bimodal distribution with two distinct peaks: a low-elasticity peak (LEP) due to the softening of cancer cells and a high-elasticity peak (HEP) reflecting the stiffening of the surrounding matrix (Figure 6c) [26]. This pattern indicates that cancer progression involves both cell softening and matrix stiffening.

**Figure 7:**
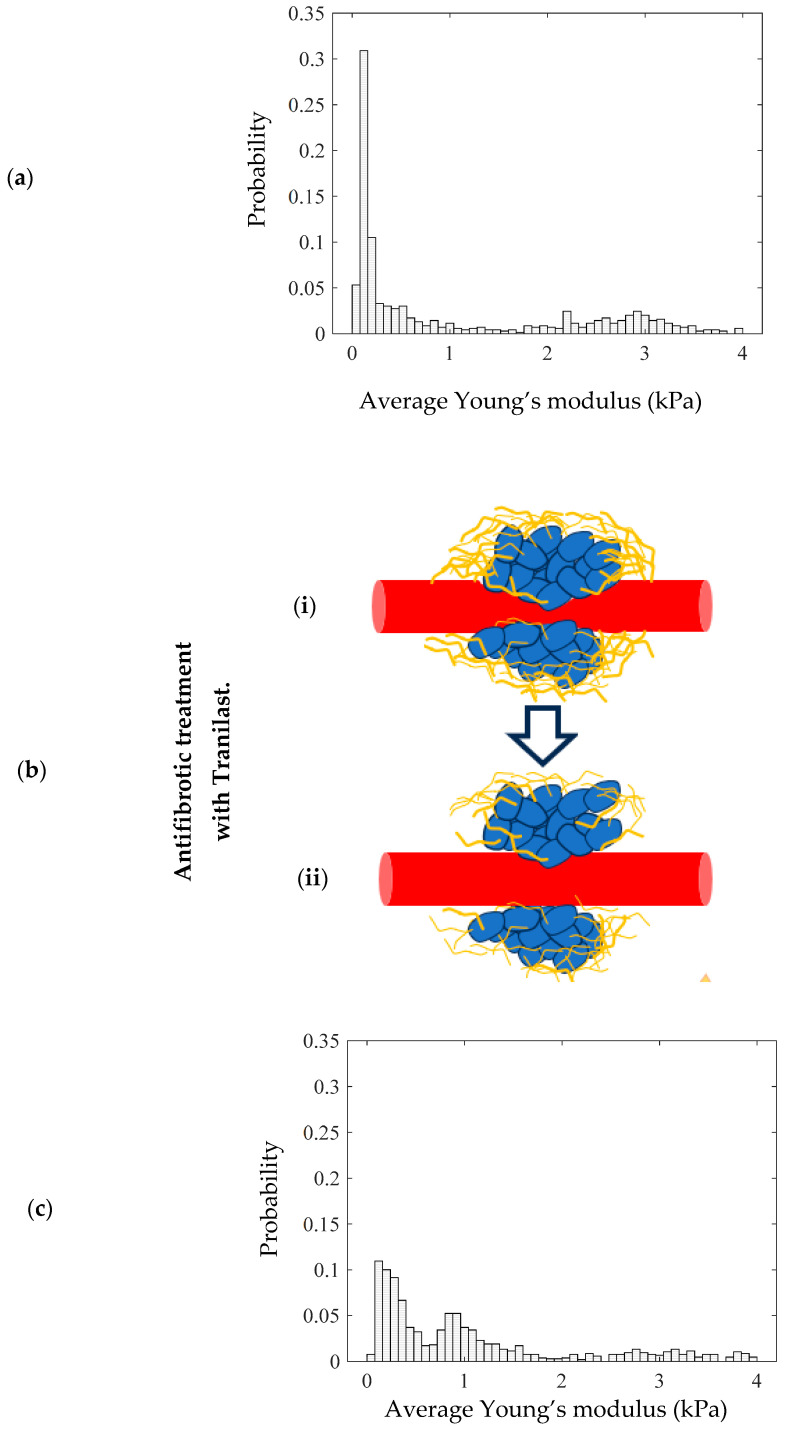
(**a**) A probability density histogram showing the average Young’s modulus distribution of the tumor. (**b**) Antifibrotic treatment with Tranilast reduces the level of collagen surrounding the tumor, altering the mechanical distribution. (**i**) In the initial condition, the tumor contains a high amount of collagen. (**ii**) Tranilast reduces the level of collagen. (**c**) A probability density histogram of a tissue section after the use of Tranilast.

A typical example of how the probability density histograms can be used to mathematically model the mechanical properties of tumor tissues and assess the effectiveness of therapeutic approaches is presented in Figure 7. To illustrate this example, we used a dataset from our previous publication [36]. This dataset included various murine tumor models subjected to different treatments. Tissue biopsies were collected at multiple time points and analyzed using AFM. The protocol for tumor establishment is analytically described in [36].

In particular, the mechanical distribution for animal tumor models (breast cancer in mice) is presented in Figure 7a. More specifically, in Figure 7a, the two-peak distribution is clearly presented. Among other therapeutic approaches in reference [36], the antifibrotic drug Tranilast is used. Tranilast has shown potential as an adjunctive therapy in tumor treatment, primarily through its antifibrotic properties (Figure 7b). Tumor-associated fibrosis, characterized by excessive collagen deposition in the tumor microenvironment, can hinder drug delivery and promote tumor progression.

By inhibiting fibroblast proliferation and reducing collagen synthesis, Tranilast helps to modify the stromal architecture, enhancing the efficacy of chemotherapy and immunotherapy. Additionally, its ability to suppress TGF-β (Transforming Growth Factor Beta) signaling and promote collagen degradation can help in reducing the desmoplastic reaction, potentially improving the overall treatment response and patient outcomes in cancer therapy. In Figure 7c, the mechanical distribution of a tumor tissue biopsy after a 28-day treatment with Tranilast is presented.

The alterations in the mechanical distribution compared to the case in Figure 7a are evident. In particular, the level of collagen is significantly lower after the treatment, leading to the displacement of the “high” average Young’s modulus peak (HEP) to the left. The main conclusion of this example is that mathematical modeling through probability density histograms is not only useful for the categorization of tissue as normal, benign, or malignant, but can also be used to evaluate the effectiveness of various therapeutic procedures.

Table 2 summarizes key studies that utilize mechanical property measurements as a means of cancer diagnosis and prognosis through AFM indentation experiments on tissue samples. In addition to detailing the types of tissues examined, the table specifies the contact mechanics models employed.

### 3.2. Novel AFM Approaches in Cancer Treatment Monitoring: Focus on Photodynamic Therapy

AFM enables objective classification of tissues and can monitor treatment responses by tracking changes in mechanical properties. A characteristic example is photodynamic therapy (PDT), a promising option for superficial tumors, which alters the tumor microenvironment and tissue mechanics. AFM can be used to monitor these changes during PDT, either alone or in combination with pharmaceutical treatments.

Photodynamic therapy (PDT) uses a photosensitizing agent, activated by localized light (laser or LEDs), to produce reactive oxygen species that destroy malignant cells [95]. PDT specifically targets cancer cells, sparing healthy tissue. It is less invasive than surgery and causes fewer side effects than chemotherapy or radiation, with shorter treatment times. Unlike radiotherapy, it has no lifetime dose limit and allows for high selectivity through localized light activation and drug delivery [96]. The U.S. Food and Drug Administration has approved several agents for PDT, including porfimer sodium, aminolevulinic acid, and methyl aminolevulinate [97].

In addition to approved drugs, many clinical trials are investigating new photosensitizers (PSs) for safety and efficacy. However, it should be noted that photodynamic therapy (PDT) may cause temporary side effects, such as skin sensitivity to light and vision changes [98]. In addition, a major limitation of PDT is reduced efficiency due to tumor microenvironment (TME) barriers, such as compressed blood vessels that hinder PS delivery. Various strategies have been developed to target the TME at the cellular, molecular, and mechanical levels [99].

Biomodulation enhances PS accumulation by regulating cell metabolism, while functional targeting addresses tumor-specific conditions like hypoxia, abnormal vasculature, and acidity. PDT itself can induce hypoxia by reducing perfusion and consuming oxygen [100], and some PSs are designed to activate under such conditions [101]. Combining photodynamic therapy (PDT) with hypoxia-reducing treatments can enhance outcomes [102,103,104,105]. Abnormal tumor vasculature supports enhanced permeability and retention (EPR), aiding drug delivery; thus, nanoparticles, liposomes, and other photosensitizer (PS) carriers are used to exploit this effect [106]. Tumor acidity is also targeted using pH-sensitive PSs [107,108]. Targeted delivery strategies, such as photoimmunotherapy, use PS–antibody conjugates to selectively treat malignant cells and cancer-associated fibroblasts (CAFs) [109,110].

AFM has been employed to evaluate cellular and structural changes induced by photodynamic therapy (PDT). In 2007, Tomankova and Bajgar used AFM to study human melanoma cells (G361) treated with the photosensitizer ZnTPPS_4_ and exposed to LED irradiation. Before PDT, cells exhibited smooth surfaces without protrusions, while post-treatment surfaces became rough, with cleaved areas and protrusions [111]. In 2009, Jung et al. applied AFM to assess cytoskeletal changes in bladder cancer cells treated with the chlorin-based compound DH-II-24. PDT caused cell shrinkage and membrane blebbing, and AFM detected ultrathin, helical microfilaments undetectable by confocal microscopy, demonstrating AFM’s enhanced sensitivity [112].

Recent studies continue to highlight AFM’s utility in monitoring cytoskeletal changes during photodynamic therapy (PDT). In 2022, Taninaka et al. combined AFM with complementary methods to investigate actin (A-filament) and stress fiber (S-fiber) formation during PDT. They found that RhoA activation occurred rapidly but stabilized early, while A-filament production persisted. AFM confirmed increased cell stiffness due to S-fiber development [113]. AFM has also been used to compare different photosensitizers and their effects during PDT. Kamiyanagi et al. (2022) examined porphylipoprotein (PLP) and talaporphyrin sodium (NPe6), finding that NPe6 accumulates in lysosomes, generates reactive oxygen species (ROS), and promotes stress fiber formation, while PLP localizes in phagosomes, delays ROS release, induces early membrane blebbing, and increases cell elasticity without RhoA activation [114]. In another study, Zhang et al. used AFM to assess the stiffness of PDT-subjected cells. Nanomaterial-based PDT using graphdiyne oxide (GDYO) as the photosensitizer increased the stiffness of oral squamous cell carcinoma (OSCC) cells, enhancing immune responses; in vivo experiments in a murine model further supported GDYO’s therapeutic potential [115]. In addition, a recent study employing atomic force microscopy (AFM), along with optical and scanning electron microscopy, reported for the first time variations in membrane stiffness of human liver (HepG2) cancer cells treated with self-assembling peptides used as a PDT nanodrug [116].

Beyond evaluating cellular responses, AFM also aids in photosensitizer development. Sukhanova et al. (2013) used AFM to examine hybrid nanosystems with zinc selenide (ZnSe) nanoparticles for PDT, demonstrating its effectiveness in analyzing nanosystem size and morphology [117]. AFM is an emerging tool in PDT research, primarily used to evaluate PDT effects in vitro, mostly as an imaging technique. However, its potential extends to characterizing tissue specimens, such as biopsies from treated tumors. Further studies are needed to fully harness AFM’s capabilities in this field.

**Figure 8:**
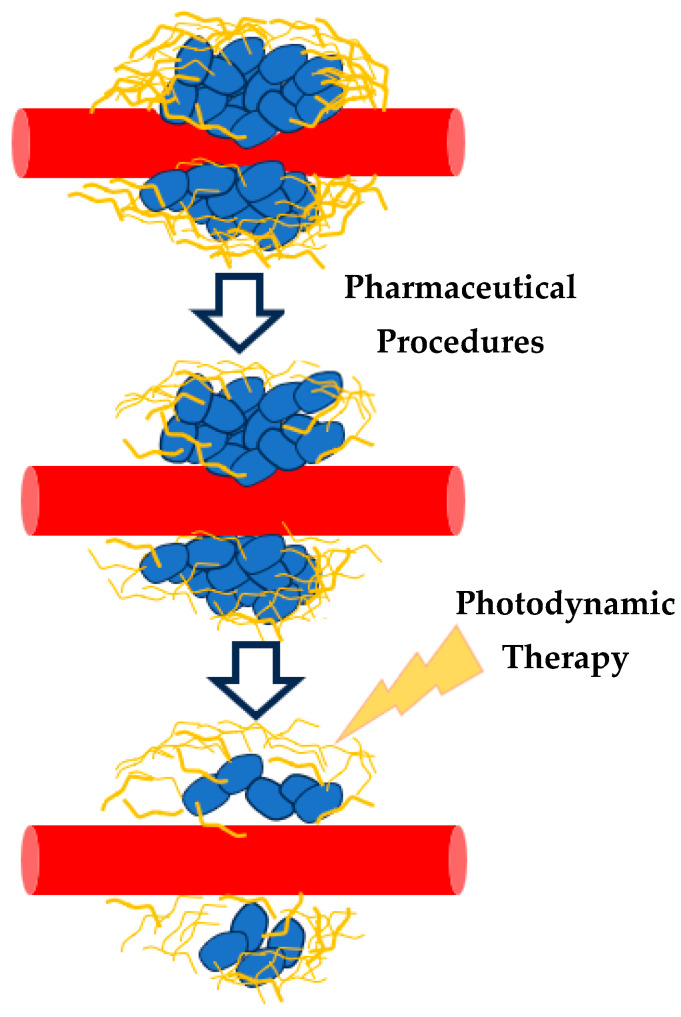
The combination of drugs (antifibrotic treatment) and PDT may lead to new, effective cancer treatment procedures. The effectiveness of these procedures can be recorded using AFM and the average Young’s modulus probability functions.

It is important to note that a successful PDT should shift tissue nanomechanical property distributions from “multi-peak” to “single-peak” mechanical property distributions [36], suggesting a promising mathematical framework for cancer treatment evaluation. This approach also supports exploring combinations of PDT with pharmaceutical agents like antifibrotics (Figure 8) [36].

## 4. Discussion and Conclusions

This review highlights recent advancements in data processing for the AFM nanoindentation method, emphasizing the use of “apparent” or “average” Young’s modulus distributions in cancer diagnosis and treatment evaluation. The potential of these techniques to provide valuable insights into the mechanical properties of tissues offers new opportunities for more accurate and personalized cancer care. Young’s modulus is a crucial physical property for characterizing the mechanical behavior of soft biological samples, such as cells, at the nanoscale. In cancer diagnosis, AFM experiments use Young’s modulus as a mechanical biomarker to differentiate between normal and cancerous cells by examining the distribution of the Young’s modulus across nanoregions. The reason is that cancer progression alters the mechano-cellular phenotype, affecting the tumor microenvironment (TME). In addition, cancer cells appear to be “softer” compared to normal ones. Despite understanding the mechanical behavior of both normal and cancer cells, achieving a user-independent, reproducible categorization of cells as normal or cancerous remains challenging. This is because AFM nanoindentation data are generally interpreted using classical applied mechanics models, with Hertzian theory being the most commonly employed for analyzing force–indentation measurements in biological samples. The Hertzian model relates the applied force and indentation depth and is often adjusted for various indenter geometries, such as conical, parabolic, or pyramidal tips, depending on the specific AFM setup and sample properties. However, the standard Hertzian model assumes that the sample behaves as a linear elastic half-space, which may not always be the case in real-world applications, particularly for soft and thin samples such as biological cells. To address this, several modifications have been proposed to account for the finite thickness of the sample and its mechanical heterogeneity. These adjustments, based on polynomial corrections and the use of generalized physical magnitudes such as the “average” Young’s modulus values or functions, aim to improve the accuracy of the measurements. In particular, they seek to account for the depth-dependent mechanical properties that classic theories do not consider. Therefore, 3D mechanical nanocharacterization is also possible, enhancing the ability to easily obtain reproducible results across different research groups.

Apart from issues related to the sample’s finite thickness and heterogeneity in obtaining user-independent and reproducible results, the accuracy of AFM measurements on biological samples can be affected by imperfections in the conical shape of the AFM tip. Errors arise from the tip apex, which may deviate from the ideal shape, leading to an overestimation of the “apparent” or “average” Young’s modulus. The force applied to the sample is influenced by this imperfection and is described by a more complex equation than Sneddon’s equation for perfect conical indenters, which includes additional terms related to the tip shape. Using the classic Sneddon model, which assumes a perfect conical indenter, can result in inaccurate measurements of mechanical properties.

It is also important to highlight that biological samples also exhibit viscoelasticity, meaning they show both elastic and viscous behavior. In particular, living cells exhibit complex viscoelastic behaviors closely linked to their functions in both physiological and pathological conditions. Recently, interesting models have been introduced in the literature to capture the viscoelastic behavior of cells [118]. A universal two-stage power-law rheology has been observed in all cell types and tissues, suggesting a fundamental rheological characteristic inherent to biological systems [119]. Viscoelasticity causes a difference between loading and unloading curves due to energy dissipation during deformation. Moreover, the stiffness of these materials varies with the loading rate, influencing the measured mechanical properties. To minimize these effects and ensure reproducible results, many studies use low loading rates, particularly in cancer diagnosis protocols where samples are approximated as elastic. This is the reason for employing Hertzian mechanics models for data processing.

Regarding the categorization of tissue samples as normal, benign or malignant, a quantitative method involving frequency histograms (probability density histograms) of the average Young’s modulus values has been proposed. The method applies distributions of mechanical properties (“one-peak”, “two-peak”, or “multi-peak”) to represent tissue stiffness, allowing for tissue classification and assessment of therapies that modify the tumor’s mechanical properties. Although individual cancer cells are generally softer than their normal counterparts, it is widely acknowledged that tumors as a whole exhibit increased stiffness compared to the surrounding healthy tissue. This apparent contradiction arises because the overall mechanical properties of a tumor are not determined solely by the cancer cells themselves but are strongly influenced by the tumor microenvironment, particularly the stromal tissue. The stroma undergoes extensive remodeling during tumor progression, characterized by increased deposition and crosslinking of extracellular matrix components such as collagen. This remodeling results in a denser and mechanically stiffer matrix, which contributes significantly to the overall stiffness of the tumor mass. 

In addition, it has been demonstrated that antifibrotic drugs, such as Tranilast, can significantly change the mechanical properties of tissue sections (Figure 7). This approach demonstrates the potential of AFM and mathematical modeling for assessing cancer progression and treatment efficacy. In addition, the mathematical modeling presented in this paper can evaluate new therapeutic procedures that combine existing ones to improve their efficacy. A typical case is the use of photodynamic therapy in combination with drugs. Given the PDT- and drug-induced mechanical changes in tissues, AFM could serve as a powerful tool for quantitatively evaluating the effectiveness of combined treatments. By examining nanomechanical property distributions before and after treatment, AFM can contribute to optimizing therapeutic strategies and advancing personalized cancer care. Moreover, integrating PDT with antifibrotic drugs or other pharmaceutical interventions offers a promising direction for future cancer therapies, further extending AFM’s significance in both research and clinical settings.

## Figures and Tables

**Table 1:** Fundamental equations for processing AFM data on soft samples. F: applied force, h: indentation depth, E: Young’s modulus, v: Poisson’s ratio, θ: cone’s half angle, R: tip radius, $r_{c}$: contact radius, $h_{c}$: contact depth, $c_{M}$: fitting coefficients depending on the h/R ratio [50], H: sample’s thickness, γ: surface energy density, E(c): average Young’s modulus, A,ξ,C: fitting coefficients determined using multiple indentations on the same location [60,64], $h_{T}$*:* transition depth, $R_{T}$: transition radius, B, n: arbitrary constants depending on the tip geometry, Γ: gamma function.

Assumptions	Tip Geometry	Force–Indentation Equation
Elastic half-space	Conical indenter	$F=\frac{2}{\pi }\frac{Ε}{1-v^{2}}\tan\left(\theta \right)h^{2}$
	Four-sided pyramidal tip	$F=\frac{1}{\sqrt{2}}\frac{Ε}{1-v^{2}}\tan\left(\theta \right)h^{2}$
	Parabolic indenter	$F=\frac{4}{3}\frac{Ε}{1-v^{2}}R^{1/2}h^{3/2}$
	Spherical indenter	$F=\frac{E}{2\left(1-v^{2}\right)}\left[\left(r_{c}^{2}+R^{2}\right)ln\left(\frac{R+r_{c}}{R-r_{c}}\right)-2r_{c}R\right]\;and\;ln\left(\frac{R+r_{c}}{R-r_{c}}\right)=\frac{2h}{r_{c}}$ or, $F=\frac{4ER^{\frac{1}{2}}}{3\left(1-v^{2}\right)}h^{\frac{3}{2}}\left[c_{1}+\sum_{M=2}^{N}\frac{3}{2Μ}c_{M}R^{\left(\frac{3}{2}-M\right)}h^{M-\frac{3}{2}}\right]$
	General nanoindentation equation	$E=\frac{\sqrt{\pi }}{2}\left(1-v^{2}\right)\frac{S}{\sqrt{A}},\;\;\;\;where:S={\left.\frac{dF}{dh}\right|}_{h_{max}}$ $\mathrm{Parabolic}\;\mathrm{indenters}:\;A=\pi R{h\;}_{max}$ $\mathrm{Spherical}\;\mathrm{indenters}:\;A=\pi \sqrt{2R{h\;}_{c}-h_{c}^{2}\;}$ $\mathrm{Conical}\;\mathrm{indenters}:\;A=\pi h_{c}^{2}\tan^{2}(\theta )$
Thin samples (v=0.5)	Conical indenter	$F_{cone}=\frac{8}{3\pi }E\tan\left(\theta \right)h^{2}\left[1+\frac{0.721htan(\theta )}{H}+\frac{0.650h^{2}\tan^{2}\left(\theta \right)}{H^{2}}+\frac{0.491h^{3}\tan^{3}\left(\theta \right)}{H^{3}}+\frac{0.225h^{4}\tan^{4}\left(\theta \right)}{H^{4}}\right]$
	Parabolic indenter	$F_{paraboloid}=\frac{16}{9}ER^{\frac{1}{2}}h^{\frac{3}{2}}\left[1+\frac{1.133\sqrt{Rh}}{H}+\frac{1.497Rh}{H^{2}}+\frac{1.469{\left(Rh\right)}^{\frac{3}{2}}}{H^{3}}+\frac{0.755{\left(Rh\right)}^{2}}{H^{4}}\right]$
Heterogeneous samples	Conical indenter	$F=\frac{2}{\pi }\frac{E}{1-v^{2}}\tan(\theta )h^{2}\left[1+a_{C}{\left(\frac{s}{htan(\theta )}\right)}^{\beta_{C}}\right]$ where, $s=2\gamma (1-v^{2})/E$, $a_{C}=0.95\pm 0.0097$ and $\beta_{C}=0.92\pm 0.0088$. or, $F=\frac{2}{\pi }\frac{E\left(c\right)}{1-v^{2}}\tan(\theta )h^{2}$ where, $E\left(c\right)=Ah^{\xi }+C\;\;\;(A,C>0,\;\xi <0)$
	Parabolic indenter	$F=\frac{4}{3}\frac{E}{1-v^{2}}R^{1/2}h^{3/2}\left[1+a_{s}{\left(\frac{s}{\sqrt{Rh}}\right)}^{\beta_{s}}\right]$ where, $s=2\gamma (1-v^{2})/E$, $a_{s}=0.88\pm 0.0037$ and $\beta_{s}=0.87\pm 0.0034.$ or, $F=\frac{4}{3}\frac{Ε(c)}{1-v^{2}}R^{1/2}h^{3/2}$ where, $E\left(c\right)=Ah^{\xi }+C\;\;\;(A,C>0,\;\xi <0)$
Complicated tip geometries	Tip apex imperfections	Conical with round tip apex: $F=\frac{2}{\pi }\frac{E}{1-v^{2}}\tan\left(\theta \right)h^{2}+\frac{4}{\pi }\frac{E}{1-v^{2}}[R_{T}-\tan\left(\theta \right)h_{T}]h\;$ Truncated cone: $F=\frac{2}{\pi }\frac{E}{1-v^{2}}\tan\left(\theta \right)h^{2}+\frac{4}{\pi }\frac{E}{1-v^{2}}R_{T}h\;$ Generic shape of tip apex ($f\left(r\right)=Br^{n}$): $F=\frac{2}{\pi }\frac{E}{1-v^{2}}\tan\left(\theta \right)h^{2}+\frac{4}{\pi }\frac{E}{1-v^{2}}\left\{\frac{1}{{\left(\sqrt{\pi }Β\right)}^{1/n}}{\left[\frac{\Gamma \left(\frac{n}{2}+\frac{1}{2}\right)}{\Gamma \left(\frac{n}{2}+1\right)}\right]}^{1/n}\;h_{T}^{1/n}-h_{T}\tan\left(\theta \right)\right\}h$

Note: The equations above can be extended to heterogeneous samples. In this case, the average Young’s modulus *E*(*c*), is calculated for a specific indentation depth.

**Table 2:** Key AFM studies on cancer research using tissue indentation experiments.

Work	Tissue Sample	Tip Geometry	Model	Force–Indentation Equation
Plodinec et al. (2012) [26]	Normal, benign, and malignant breast tissue	Pyramidal (conical approximation)	Oliver and Pharr	$E=\frac{\sqrt{\pi }}{2}\left(1-v^{2}\right)\frac{S}{\sqrt{A}}$, $A=4h^{2}\tan^{2}(\theta )$
Tian et al. (2015) [30]	Liver cancer tissues	Pyramidal (conical approximation)	Sneddon	$F=\frac{2}{\pi }\frac{Ε}{1-v^{2}}\tan\left(\theta \right)h^{2}$
Ciasca et al. (2016) [31]	Brain tumor	Pyramidal (conical approximation)	Sneddon	$F=\frac{2}{\pi }\frac{Ε}{1-v^{2}}\tan\left(\theta \right)h^{2}$
Chen et al. (2021) [93]	Breast cancer bone metastases	Spherical	Hertz/Sneddon	$F=\frac{4}{3}\frac{Ε}{1-v^{2}}R^{\frac{1}{2}}h^{\frac{3}{2}}\;\;\;and$ $F=\frac{E}{2\left(1-v^{2}\right)}\left[\left(r_{c}^{2}+R^{2}\right)ln\left(\frac{R+r_{c}}{R-r_{c}}\right)-2r_{c}R\right]\;\;\;with$ $\ln\left(\frac{R+r_{c}}{R-r_{c}}\right)=\frac{2h}{r_{c}}$
Levillain et al. (2022) [39]	Breast, kidney, and thyroid tumors	Spherical	Hertz/Sneddon	$F=\frac{4}{3}\frac{Ε}{1-v^{2}}R^{\frac{1}{2}}h^{\frac{3}{2}}\;\;\;\;and$ $F=\frac{E}{2\left(1-v^{2}\right)}\left[\left(r_{c}^{2}+R^{2}\right)ln\left(\frac{R+r_{c}}{R-r_{c}}\right)-2r_{c}R\right]\;\;\;with$ $\ln\left(\frac{R+r_{c}}{R-r_{c}}\right)=\frac{2h}{r_{c}}$
Stylianou et al. (2022) [36]	Breast and fibrosarcoma tumors	Pyramidal (conical approximation)	Sneddon	$F=\frac{2}{\pi }\frac{Ε}{1-v^{2}}\tan\left(\theta \right)h^{2}$
Stylianou et al. (2023) [34]	Normal pancreatic tissue and pancreatic tumor tissue	Pyramidal (conical approximation)	Sneddon	$F=\frac{2}{\pi }\frac{Ε}{1-v^{2}}\tan\left(\theta \right)h^{2}$

## Data Availability

No new data were created or analyzed in this study. Data sharing is not applicable to this article.
